# PRKAR2B‐HIF‐1α loop promotes aerobic glycolysis and tumour growth in prostate cancer

**DOI:** 10.1111/cpr.12918

**Published:** 2020-10-07

**Authors:** Lei Xia, Jian Sun, Shaowei Xie, Chenfei Chi, Yinjie Zhu, Jiahua Pan, Baijun Dong, Yiran Huang, Weiliang Xia, Jianjun Sha, Wei Xue

**Affiliations:** ^1^ Department of Urology Ren Ji Hospital School of Medicine Shanghai Jiao Tong University Shanghai China; ^2^ Department of Urology Affiliated Hospital of Jiangnan University Jiangsu China; ^3^ Department of Ultrasound Renji Hospital School of Medicine Shanghai Jiao Tong University Shanghai China; ^4^ School of Biomedical Engineering Shanghai Jiao Tong University Shanghai China

**Keywords:** aerobic glycolysis, glycolytic ability, HIF1A, PRKAR2, RII‐BETA

## Abstract

**Objectives:**

Reprogramming of cellular metabolism is profoundly implicated in tumorigenesis and can be exploited to cancer treatment. Cancer cells are known for their propensity to use glucose‐dependent glycolytic pathway instead of mitochondrial oxidative phosphorylation for energy generation even in the presence of oxygen, a phenomenon known as Warburg effect. The type II beta regulatory subunit of protein kinase A (PKA), PRKAR2B, is highly expressed in castration‐resistant prostate cancer (CRPC) and contributes to tumour growth and metastasis. However, whether PRKAR2B regulates glucose metabolism in prostate cancer remains largely unknown.

**Materials and methods:**

Loss‐of‐function and gain‐of‐function studies were used to investigate the regulatory role of PRKAR2B in aerobic glycolysis. Real‐time qPCR, Western blotting, luciferase reporter assay and chromatin immunoprecipitation were employed to determine the underlying mechanisms.

**Results:**

PRKAR2B was sufficient to enhance the Warburg effect as demonstrated by glucose consumption, lactate production and extracellular acidification rate. Mechanistically, loss‐of‐function and gain‐of‐function studies showed that PRKAR2B was critically involved in the tumour growth of prostate cancer. PRKAR2B was able to increase the expression level of hypoxia‐inducible factor 1α (HIF‐1α), which is a key mediator of the Warburg effect. Moreover, we uncovered that HIF‐1α is a key transcription factor responsible for inducing PRKAR2B expression in prostate cancer. Importantly, inhibition of glycolysis by the glycolytic inhibitor 2‐deoxy‐d‐glucose (2‐DG) or replacement of glucose in the culture medium with galactose (which has a much lower rate than glucose entry into glycolysis) largely compromised PRKAR2B‐mediated tumour‐promoting effect. Similar phenomenon was noticed by genetic silencing of HIF‐1α.

**Conclusions:**

Our study identified that PRKAR2B‐HIF‐1α loop enhances the Warburg effect to enable growth advantage in prostate cancer.

## Graphical abstract

Proposed model illustrates that PRKAR2B‐HIF1α loop promotes aerobic glycolysis and tumour growth in prostate cancer. On the one hand, HIF1α is a key transcription factor responsible for inducing PRKAR2B expression in prostate cancer. On the other hand, PRKAR2B was able to increase the expression level of HIF1α.

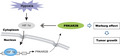

## 1 INTRODUCTION

Prostate cancer (PCa) is a leading cause of cancer‐related deaths in developed countries.^1^ While localized PCa has a favourable clinical outcome, some patients present with or develop metastatic disease. The androgen receptor plays a crucial role in prostate tumorigenesis at both early and late stages. Androgen deprivation therapy combined with bilateral orchiectomy or administration of luteinizing hormone‐releasing hormone agonists is recommended as the first‐line treatment for patients with recurrent or advanced PCa.^2^ While androgen deprivation therapy is transiently effective in advanced PCa, tumours inevitably progress to castration‐resistant prostate cancer (CRPC), which no longer responds to conventional therapies.^3^, ^4^, ^5^ Therefore, it is imperative to develop new approaches and uncover novel drug targets for the treatment of CRPC.^6^

The cyclic adenosine monophosphate (cAMP) is an important second messenger with diverse physiological functions, including cell proliferation and differentiation. Protein kinase A (PKA) is the master switch for cAMP‐mediated signalling. PKA is composed of two catalytic subunits and a cAMP‐binding regulatory subunit dimer. There are four different regulatory subunits (RIα, RIβ, RIIα and RIIβ) of PKA in human mammalian tissues.^7^ Protein kinase cAMP‐dependent regulatory type II beta (PRKAR2B) plays a role in oocyte maturation,^8^ regulates murine and human adipocyte differentiation^9^ and inhibits the effect of cAMP responsive element binding protein (CREB) activity in T cells.^10^ Recently, we identified PRKAR2B as a novel oncogene implicated in the development of CRPC.^11^, ^12^ Moreover, PRKAR2B induces the epithelial‐mesenchymal transition process and promotes prostate cancer metastasis by activating Wnt/β‐catenin signalling pathway.^13^

Reprogrammed energy metabolism is emerged as a hallmark of cancers. One of the most notable aspects of this metabolic reprogramming is aerobic glycolysis, also known as the Warburg effect. Increased Warburg effect in cancer cells is critical for tumour growth and predicts a poor prognosis in cancer patients.^14^, ^15^ In prostate cancer, androgen is sufficient to promote glucose uptake in prostate cancer. Functional genomic studies revealed that human prostate cancer cells treated with androgens showed significant cellular metabolic reprogramming, especially aerobic glycolysis.^16^ Several studies also confirmed the effect of androgen in promoting prostate cancer glycolysis.^17^, ^18^ Mechanistically, it has been reported that androgen receptor directly bound to the GLUT1 gene promoter to promote GLUT1 transcription.^19^ Moreover, the cAMP/PKA pathway is well‐documented to play a regulatory role in the glycolytic metabolism, exerting a stimulatory effect on glucose transport and utilization via phosphorylation of different downstream target proteins.^20^, ^21^ Mouse and human studies suggest that PRKAR2B may be involved in energy metabolism.^22^ Interestingly, PRKAR2B is responsible for the maturation of oocytes by controlling spindle formation and pentose phosphate pathway (PPP)‐mediated metabolism.^8^ Therefore, we reasoned whether PRKAR2B contributes to the Warburg effect to promote tumour progression in prostate cancer.

## 2 MATERIALS AND METHODS

### 2.1 Cell culture, tissue samples and reagents

The human prostate cancer cell lines (DU145, PC3 and LNCaP) were obtained from American Type Culture Collection (ATCC). LNCaP and PC3 cells were cultured in RPMI 1640 (Life Technologies), while DU145 cells were maintained in DMEM (Life technology). All prostate cancer cells were supplemented with 10% foetal bovine serum (PBS, Gibco) and 1% penicillin/streptomycin (Invitrogen) at 37°C in a humidified incubator under 5% CO_2_ condition. The LNCaP‐AI cells were generated as reported previously.^13^ Twenty primary prostate cancer tissues and 17 metastatic castration‐resistant prostate cancer (CRPC) tissues were obtained from Ren Ji Hospital, School of Medicine, Shanghai Jiaotong University.^13^ This study was approved by the institutional ethics review board of Shanghai Jiao Tong University. Galactose and 2‐deoxy‐d‐glucose (2‐DG) were purchased from Sigma‐Aldrich. The specific PKA inhibitor H89 (S1582) was obtained from Selleck.

### 2.2 Online database

The online GEPIA 2 database (http://gepia2.cancer‐pku.cn/#index) was used for correlation analysis in this study.^23^ In this study, correlation analysis was used to determine the link between PRKAR2B and glycolytic components in prostate adenocarcinoma (PRAD, n = 492) from The Cancer Genome Atlas (TCGA).

### 2.3 RNA isolation and quantitative real‐time PCR

Total RNA was extracted using TRIzol Reagent (Life Technologies) and reversely transcribed to cDNA by PrimeScript RT reagent Kit (Takara) in accordance with the manufacturer's instructions. Real‐time qPCR analyses were performed with SYBR Premix Ex Taq (Takara) on ABI 7300 detection system. PCR primer sets were designed using Primer Premier 5, and the sequences were as follows: PRKAR2B, 5′‐TTCGGCGAACTGGCCTTAATG‐3′ (forward) and 5′‐ACTTTCAGGCGTTCAGAAAACT‐3′ (reverse); SLC2A1, 5′‐ATTGGCTCCGGTATCGTCAAC‐3′ (forward) and 5′‐GCTCAGATAGGACATCCAGGGTA‐3′ (reverse); PFKP, 5′‐CGCCTACCTCAACGTGGTG‐3′ (forward) and 5′‐ACCTCCAGAACGAAGGTCCTC‐3′ (reverse); PKM, 5’‐ATGTCGAAGCCCCATAGTGAA‐3′ (forward) and 5′‐TGGGTGGTGAATCAATGTCCA‐3′ (reverse); LDHA, 5′‐ATGGCAACTCTAAAGGATCAGC‐3′ (forward) and 5′‐CCAACCCCAACAACTGTAATCT‐3′ (reverse); β‐actin, 5′‐CGTCATACTCCTGCTTGCTG‐3′ (forward) and 5′‐GTACGCCAACACAGTGCTG‐3′ (reverse).

### 2.4 Short hairpin RNA and cell transfection

Short hairpin RNA against PRKAR2B used in this study was synthesized by GenePharma. Transfection of the oligonucleotide duplexes was performed in a 6‐well plate (1 × 10^5^ cells per well) with Lipofectamine 2000 (Invitrogen) according to the manufacturer's instructions. Puromycin dihydrochloride was used for the selection of stable clones. For overexpression strategy, 48 hours after transfection, overexpression efficiency was examined through Western blotting or real‐time qPCR analysis. For siRNA‐mediated HIF‐1α knockdown, 2 ~ 5 × 10^5^ PCa cells were plated at 6‐well plate. When cells were reached at 60%‐70% confluence, 50 ng of specific siRNA against HIF‐1α was transfected with Lipofectamine^®^ RNAiMAX reagent (Thermo Fisher Scientific). Scrambled siRNA targeting no known gene sequence was used as control. The sequences of two HIF‐1α siRNAs were shown as follows: #1, UGAUACCAACAGUAACCAAdTdT; #2, GAGGAAGAACUAAAUCCAAdTdT.

### 2.5 Luciferase reporter assay

Wild‐type PRKAR2B gene (Gene ID: 5577; NM_002736; gene location: NC_000007.14 (107044705.0.107161811) promoter was amplified from the genomic DNA and cloned into the pGL4‐Basic luciferase reporter vector (Promega). Mutated PRKAR2B promoter of predicted HIF‐1α binding sites was directly synthesized by Sangon Biotech and cloned into the pGL4‐Basic vector (Promega). All constructs were verified by sequencing. DU145 cells were seeded in 12‐well dishes and transfected with 1 μg per well WT or mutant PRKAR2B‐promoter luciferase reporter and 0.1 μg HIF‐1α expression vector using X‐tremeGENE 9 according to the manufacturer's protocol (Promega). At 48 hours post‐transfection, cell lysis was obtained, followed by analysis of firefly and renilla luciferase activities using a Dual‐Luciferase‐Reporter Assay Kit (Promega). The experiment was performed in triplicate. The ratio of firefly to Renilla luciferase activity was served as an indicator of normalized luciferase activity for each group.

### 2.6 Western blotting

Total cell lysates were prepared using a lysis buffer containing 50 mmol/L TRIS‐HCl, pH7.4, 150 mmol/L NaCl, 0.5% NP40, 50 mmol/L NaF, 1 mmol/L Na_3_VO_4_, 1 mmol/L phenyl‐methylsulfonyl fluoride, 25 μg/mL leupeptin and 25 μg/ml aprotinin and clarified by centrifugation (14 000 g for 30 min at 4°C). The protein concentration was detected by a BCA Protein Assay Kit (Pierce Biotechnology). Then, cell lysates were separated by sodium dodecyl sulphate SDS‐PAGE and transferred onto nitrocellulose membrane. The membranes were subsequently blocked with 5% (w/v) skim milk and incubated overnight with primary antibody. The next day, the membrane was incubated with second antibody at room temperature for 2 hours, washed with PBST and then developed with the ECL system. The results of Western blot were analysed with Odyssey software version 3.0. The PRKAR2B antibody (PA5‐28266) was acquired from Invitrogen. The anti‐β‐actin primary antibody was obtained from Sigma.

### 2.7 Detection of glucose and lactate level

Glucose and lactate concentration in the culture medium were measured using a Glucose Assay Kit (BioVision) and a Lactate Assay Kit (BioVision) according to the manufacturer's instructions. Conditioned cell culture media were diluted 1:100‐1:400 with assay buffer and prepared for the colorimetric assay. The absorbance was measured at 570 nm using a multifunctional microplate reader (Molecular Devices) immediately after sample preparation. The experiment was performed at least in triplicate.

### 2.8 Measurement of extracellular acidification rate and oxygen consumption rate

To detect extracellular acidification rate (ECAR) and oxygen consumption rate (OCR), the XF96 Bioenergetic Analyzers (Seahorse Bioscience) was used. ECAR and OCR were analysed using Seahorse XF Glycolysis Stress Test Kit and Seahorse XF Cell Mito Stress Test Kit, respectively. In brief, 2 × 10^4^ cells per well were seeded into a Seahorse XF 96 cell culture microplate. For ECAR measurement, glucose, oligomycin, and 2‐DG were sequentially injected into each well at indicated time points. For OCR measurement, oligomycin, FCCP (p‐trifluoromethoxy carbonyl cyanide phenylhydrazone) and rotenone plus antimycin A (R&A) were sequentially injected. Data were assessed by Seahorse XF‐96 Wave software. ECAR was shown in mpH/minute and OCR in pmols/minute.

### 2.9 HIF‐1α transcription activity assay

To determine HIF‐1α activity, the nuclear extract lysates from tested cells were harvested after genetic manipulation using the Nuclear Extraction kit (ab113474; Abcam). HIF‐1α activity was then measured by the HIF‐1αTranscription Factor Assay Kit (cat. no. ab133104; Abcam) according to the manufacturer's instructions.

### 2.10 Chromatin Immunoprecipitation

Chromatin Immunoprecipitation (ChIP) assay was performed using the Magna ChIP™ G Assay Kit according to the manufacturer's instructions. Briefly, DU145 cells overexpressing HIF‐1α or infected with control vector were used for analysis. Cells were cross‐linked with 37% formaldehyde for 10 minutes at room temperature. After washing with ice‐cold PBS for three times, cells were collected by scraping and pelleted by centrifugation at 200 g for 5 minutes. Then, fixed cells were resuspended in lysis buffer, sonicated and centrifuged to remove the insoluble material. The supernatants were incubated with indicated antibodies (HIF‐1α, ab113474, Abcam; H3K4me3, ab113474; Abcam) and Protein G magnetic beads overnight. Finally, the precipitated chromatin complexes were collected, purified and de‐cross‐linked at 62°C for 2 hours, followed by incubation at 95°C for 10 minutes. The precipitated DNA fragments were analysed by RT‐PCR analysis.

### 2.11 Plate clone formation assay

Cells were seeded into 6‐well plates with 500 or 1000 cells in each well and were cultured with 10% FBS. After 10‐14 days, the plates were washed with PBS and stained with crystal violet for 15 minutes. All experiments were performed in triplicates.

### 2.12 Cell viability and cell apoptosis assay

Cell viability assay was measured using the Cell Counting Kit‐8 kit (CCK‐8; Dojindo laboratories). Briefly, cells were seeded in 96‐well plates at a density of 5 × 10^3^ cells per well. After day 1, 3, 5, cell viability was detected by adding 10 μL of CCK‐8 solution to each well. After incubation for 1.5 hours, cell viability was measured by determining the absorbance (450 nm) using a BIO‐TEK Microplate Reader (Bio‐Tek). Cell apoptosis was induced by serum‐starvation for 48 hours and cell apoptosis assay was measured by using Apo‐ONE Caspase‐3/7 kit (Promega, G7790) and Annexin V/PI staining, respectively. All experiments were performed in triplicates. For Annexin V/PI staining, indicated PCa cells were harvested with 0.25% trypsin, centrifuged, followed by Annexin V/propidium iodide (PI) assay according to manufacturer's instructions (BD Pharmingen). The percentages of Annexin V (+) and PI (−) cells in suspension were analysed by flow cytometry.

### 2.13 Animal experiment

BALB/c nude mice (Male, 6‐week‐old) were obtained from the Chinese Academy of Sciences (Shanghai, China) and were bred and maintained in a specific pathogen‐free facility. Mice were supplied with autoclaved commercial chow and sterile water. Tumorigenicity was determined by subcutaneously injecting control or PRKAR2B‐overexpressing (LNCaP) or PRKAR2B‐silenced (DU145) cells into the flanks of nude mice (2 × 10^6^ cells). Three weeks later, the mice were killed under anaesthesia and the tumours were collected. All mouse experiments were conducted in accordance with standard operating procedures approved by the Ethical Committee of Ren Ji Hospital, School of Medicine, Shanghai Jiao Tong University.

#### 2.13.1 Statistical analysis

Data were presented as the means ± SD. Statistical analysis were performed with GraphPad Prism 5 (San Diego, CA) software or SPSS V.16.0 for Windows (IBM). Student's t test or one‐way ANOVA was used for comparisons between two groups. Correlation analysis was determined using the Spearman's test. A value of *P* < .05 was considered to be statistically significant.

## 3 RESULTS

### 3.1 PRKAR2B regulates the Warburg effect in prostate cancer

**FIGURE 1:**
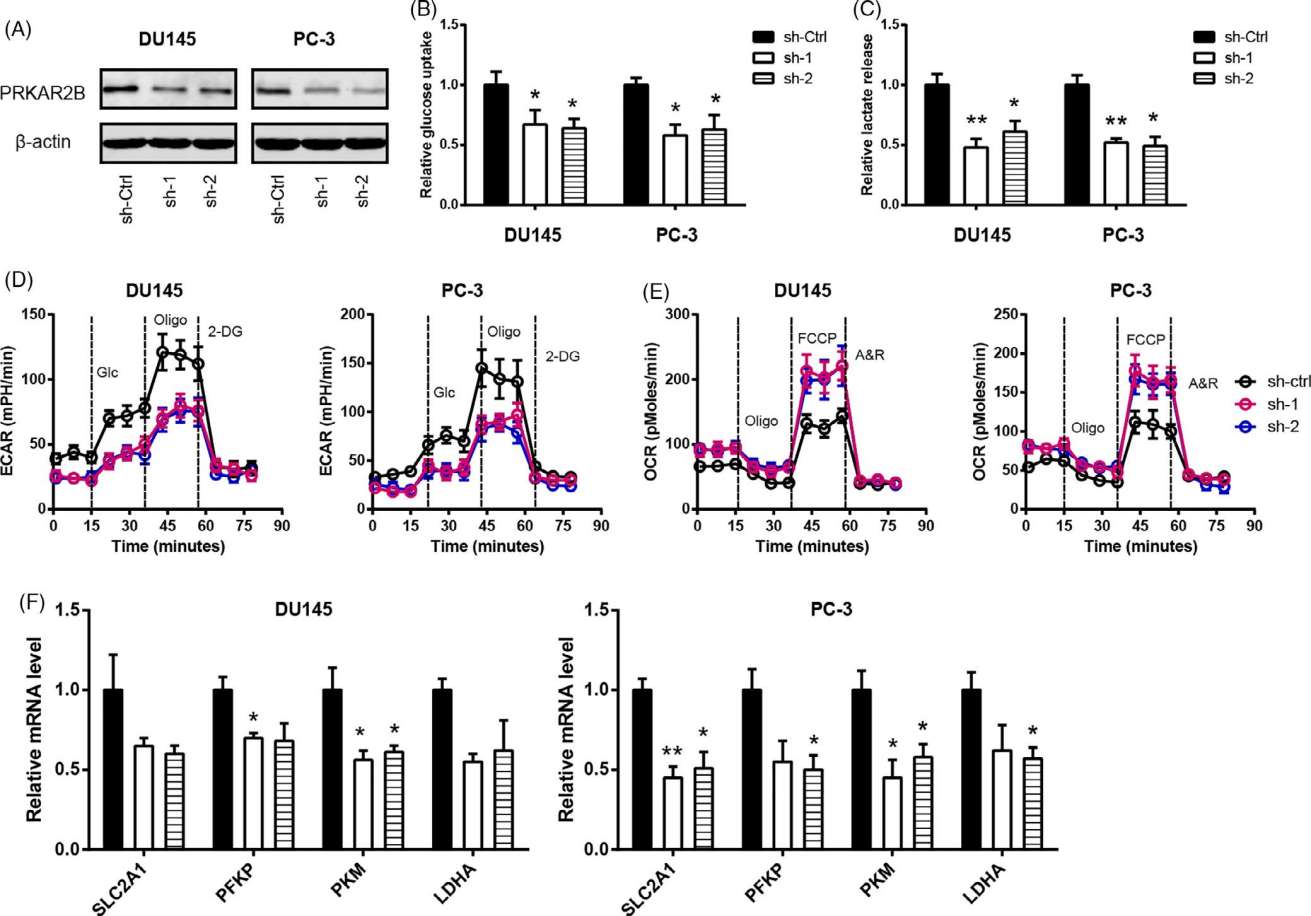
PRKAR2B knockdown inhibits prostate cancer cell glycolysis. A, Western blotting analysis of the knockdown efficiency of PRKAR2B in DU145 and PC3 cells. B‐E, The effect of PRKAR2B knockdown on the glucose utilization (B), lactate production (C), extracellular acidification rate (D), oxygen consumption rate (E), and expression of glycolytic components (F) in DU145 and PC3 cells. *P < .05; **P < .01

**FIGURE 2:**
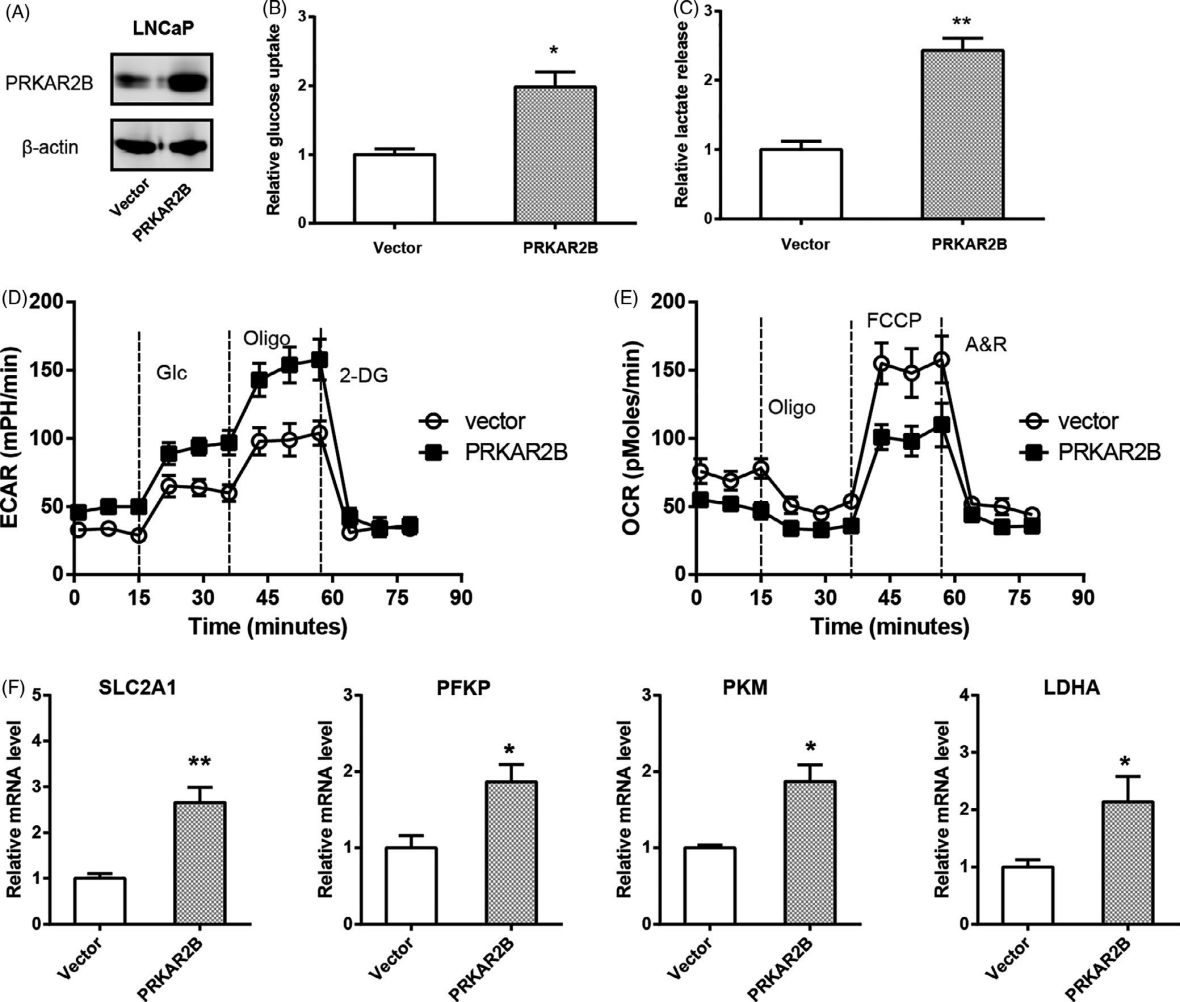
PRKAR2B overexpression promotes the Warburg effect in prostate cancer in vitro. A, Western blotting analysis of the overexpression efficiency of PRKAR2B in LNCaP cells. B‐E, The effect of PRKAR2B overexpression on the glucose utilization (B), lactate production (C), extracellular acidification rate (D), oxygen consumption rate (E), and expression of glycolytic components (F) in LNCaP cells. SLC2A1, solute carrier family 2 member 1; PFKP, phosphofructokinase platelet; PKM, pyruvate kinase muscle isozyme; LDHA, lactate dehydrogenase A. *P < .05; **P < .01

Previously, we have demonstrated that PRKAR2B is of great importance to the malignant phenotypes of prostate cancer.^11^ To determine whether PRKAR2B plays a role in the Warburg metabolism in prostate cancer, we carried out loss‐of‐function studies in two cell lines (DU145 and PC3) with high endogenous level of PRKAR2B (Figure S1). Two shRNA against PRKAR2B led to significant downregulation in PRKAR2B protein level (Figure 1A). Warburg effect is characterized by increased glucose uptake and lactate production. By detecting the glucose and lactate in the cell culture medium, we found that PRKAR2B knockdown resulted in pronounced drop in glucose utilization (Figure 1B) and lactate release (Figure 1C). To further confirm this observation, the Seahorse XF96 flux analyser showed that PRKAR2B knockdown decreased extracellular acidification rate (ECAR) (Figure 1D). As a feedback, the oxygen consumption rate (OCR) was significantly increased by PRKAR2B knockdown (Figure 1E). By real‐time qPCR analysis, we analysed the expression of glycolytic transporters and enzymes (SLC2A1, HK2, GPI, PFKP/L, ALDOA, GAPDH, PGK1, PGAM1, ENO1, PKM and LDHA). The result showed that PRKAR2B knockdown inhibited mRNA expression of glucose transporter SLC2A1 and glycolytic enzymes (phosphofructokinase platelet, pyruvate kinase muscle isozyme and lactate dehydrogenase A) (Figure 1F). Moreover, gain‐of‐function in LNCaP cells was performed to certify the role of PRKAR2B in prostate cancer cell glycolysis (Figure 2A). As shown in Figure 2B, glucose uptake in PRKAR2B‐overexpressing LNCaP cells increased to two‐fold compared to the control cells. Consistently, lactate release had a 2.5‐fold increase after PRKAR2B overexpression (Figure 2C). In addition, PRKAR2B contributed to a shift from mitochondrial oxidative phosphorylation to aerobic glycolysis as demonstrated by increased ECAR (Figure 2D) and reduced OCR (Figure 2E). Consistently, glycolytic components were increased by PRKAR2B overexpression (Figure 2F). Taken together, these findings above suggest that PRKAR2B contributes to the glycolytic phenotype in prostate cancer.

### 3.2 Androgen‐independent LNCaP cells exhibit enhanced glycolysis

**FIGURE 3:**
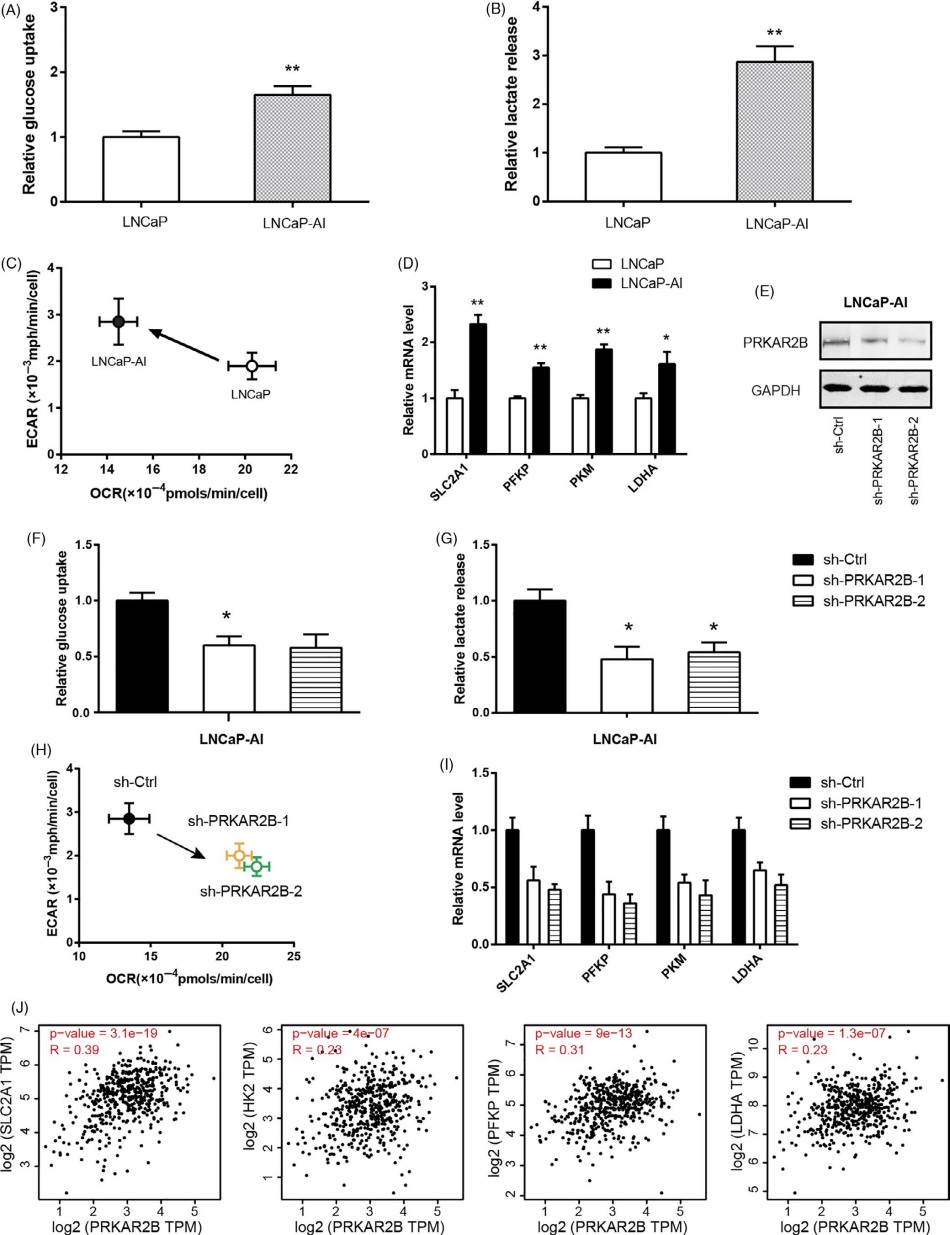
Androgen‐independent LNCaP cells exhibits enhanced glycolysis. A, The basal glucose utilization in LNCaP and LNCaP‐AI cells. B, The basal lactate production in LNCaP and LNCaP‐AI cells. C, Comparison of ECAR and OCR status in LNCaP and LNCaP‐AI cells. D, Real‐time qPCR analysis of glucose transporter and key glycolytic genes in LNCaP and LNCaP‐AI cells. E, Western blotting analysis of the effect of PRKAR2B knockdown efficiency in LNCaP‐AI cells. (F‐I) The effect of PRKAR2B knockdown on the glucose utilization (F), lactate production (G), ECAR/OCR (H), and expression of glycolytic components (I) in LNCaP‐AI cells. (J) Correlation analysis of the link between PRKAR2B expression and the expression level of glucose transporter and key glycolytic genes in prostate cancer tissues (n = 492); data were obtained from the TCGA cohort. *P < .05; **P < .01

Castration‐resistant prostate cancer (CRPC) that occurs after the failure of androgen blocking treatment causes the majority of the deaths in prostate cancer. Previously, we demonstrated that PRKAR2B expression is markedly increased in CRPC and contributes to the development of CRPC.^11^ By generating an androgen‐independent LNCaPcell line (LNCaP‐AI), we revealed that PRKAR2B promotes cell invasion in vitro and in vivo.^13^ Here, we found that LNCaP‐AI cells had increased glucose uptake (Figure 3A) and lactate production (Figure 3B) compared to its parental LNCaP cells. Moreover, Seahorse results showed that LNCaP‐AI cells exhibited higher glycolytic capacity than LNCaP cells (Figure 3C). Consistently, LNCaP‐AI cells had higher expression of the glucose transporter SLC2A1 and three key glycolytic enzymes (PFKP, PKM and LDHA) than LNCaP cells (Figure 3D). More interestingly, genetic silencing of PRKAR2B in LNCaP‐AI cells (Figure 3E) greatly suppressed its glycolytic activities, as revealed by reduced glucose uptake (Figure 3F), lactate release (Figure 3G), ECAR/OCR (Figure 3H) and expression of glycolytic components (Figure 3I). By data mining the PCa samples from TCGA cohort, we found that PRKAR2B expression was closely correlated with the expression of these glycolytic components in prostate cancer (Figure 3J) and this correlation was absent in the non‐tumour samples obtained from the Genotype‐Tissue Expression (GTEx) database (Figure S2).

### 3.3 PRKAR2B regulates HIF‐1α expression in prostate cancer

**FIGURE 4:**
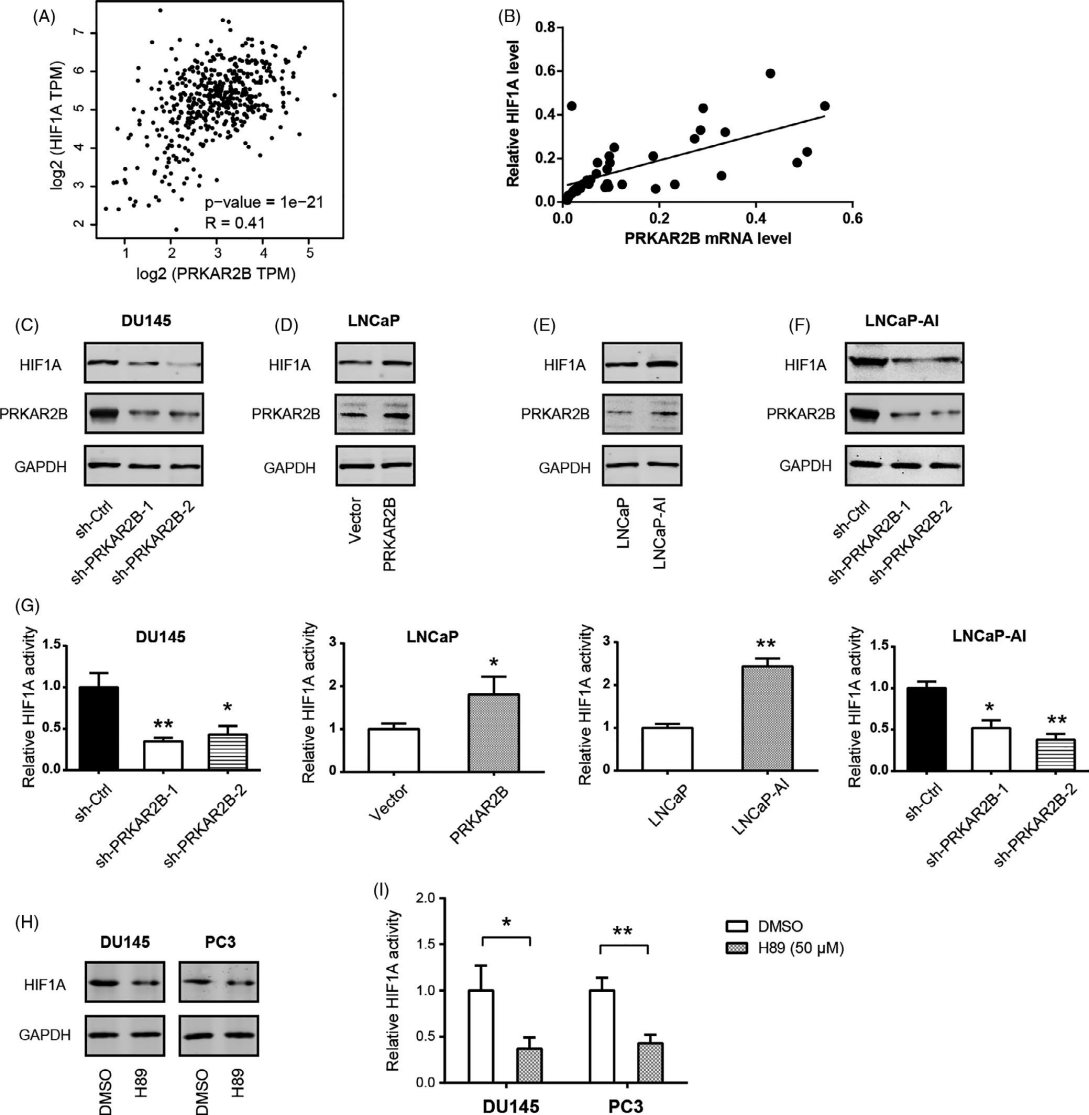
PRKAR2B regulates HIF1α expression in prostate cancer. (A‐B) Correlation analysis of the link between PRKAR2B and HIF1A expression in prostate cancer tissues; data were obtained from the TCGA cohort (A) and Ren Ji cohort (B). (C) Western blotting analysis of the effect of PRKAR2B knockdown on the HIF1A protein level in DU145 cells. (D) Western blotting analysis of the effect of PRKAR2B overexpression on the HIF1A protein level in LNCaP cells. (E) Western blotting analysis of HIF1A protein level in LNCaP and LNCaP‐AI cells. (F) Western blotting analysis of the effect of PRKAR2B knockdown on the HIF1A protein level in LNCaP‐AI cells. (G) Effects of PRKAR2B knockdown or overexpression on the HIF‐1α activity. (H) Western blotting analysis of HIF1A protein level in DU145 and PC3 cells upon H89 (50 μmol/L) treatment for 24 h. (I) Determining the effect of H89 (50 μmol/L) treatment on the HIF‐1α activity in DU145 and PC3 cells. *P < .05; **P < .01

HIF‐1α is a crucial transcriptional factor responsible for the Warburg effect and plays key roles in driving prostate tumorigenesis. Therefore, we investigated whether PRKAR2B regulates HIF‐1α expression or activity in prostate cancer. Firstly, we found a close correlation between PRKAR2B and HIF‐1α mRNA expression in the TCGA cohort (Figure 4A, *R* = 0.41). Then, we evaluated their expression in 20 primary PCa tissues and 17 metastatic CRPC tissues using real‐time qPCR. Representative IHC staining of PRKAR2B in these tissues was shown in Figure S3. As a result, mRNA expression level of PRKAR2B was positively associated with HIF‐1α expression (Figure 4C, *R* = 0.65). Genetic silencing of PRKAR2B led to significant downregulation in HIF‐1α protein level in DU145 cells (Figure 4C), while overexpression of PRKAR2B upregulated HIF‐1α protein level in LNCaP cells (Figure 4D). LNCaP‐AI cells had higher endogenous HIF‐1α level than LNCaP cells (Figure 4E). In contrast, knockdown of PRKAR2B in LNCaP‐AI cells reduced HIF‐1α protein level (Figure 4F). To further confirm the regulatory role of PRKAR2B on HIF‐1α, we detected the transcriptional activity upon genetic manipulation of PRKAR2B. Expectedly, HIF‐1α activity was reduced by PRKAR2B knockdown, while increased by PRKAR2B overexpression (Figure 4G) in PCa cells. Furthermore, we determined HIF‐1α expression upon treatment of a specific PKA inhibitor H89 for 24 hours. As a result, H89 (50 μmol/L) significantly inhibited HIF‐1α expression (Figure 4H), HIF‐1α transcriptional activity (Figure 4I), glucose uptake (Figure S4A), and lactate production (Figure S4B) in DU145 and PC3 cells. Collectively, these findings suggest that PRKAR2B might promote glycolysis by regulating HIF‐1α in prostate cancer.

### 3.4 Transcriptional regulation of PRKAR2B by HIF‐1α in prostate cancer

**FIGURE 5:**
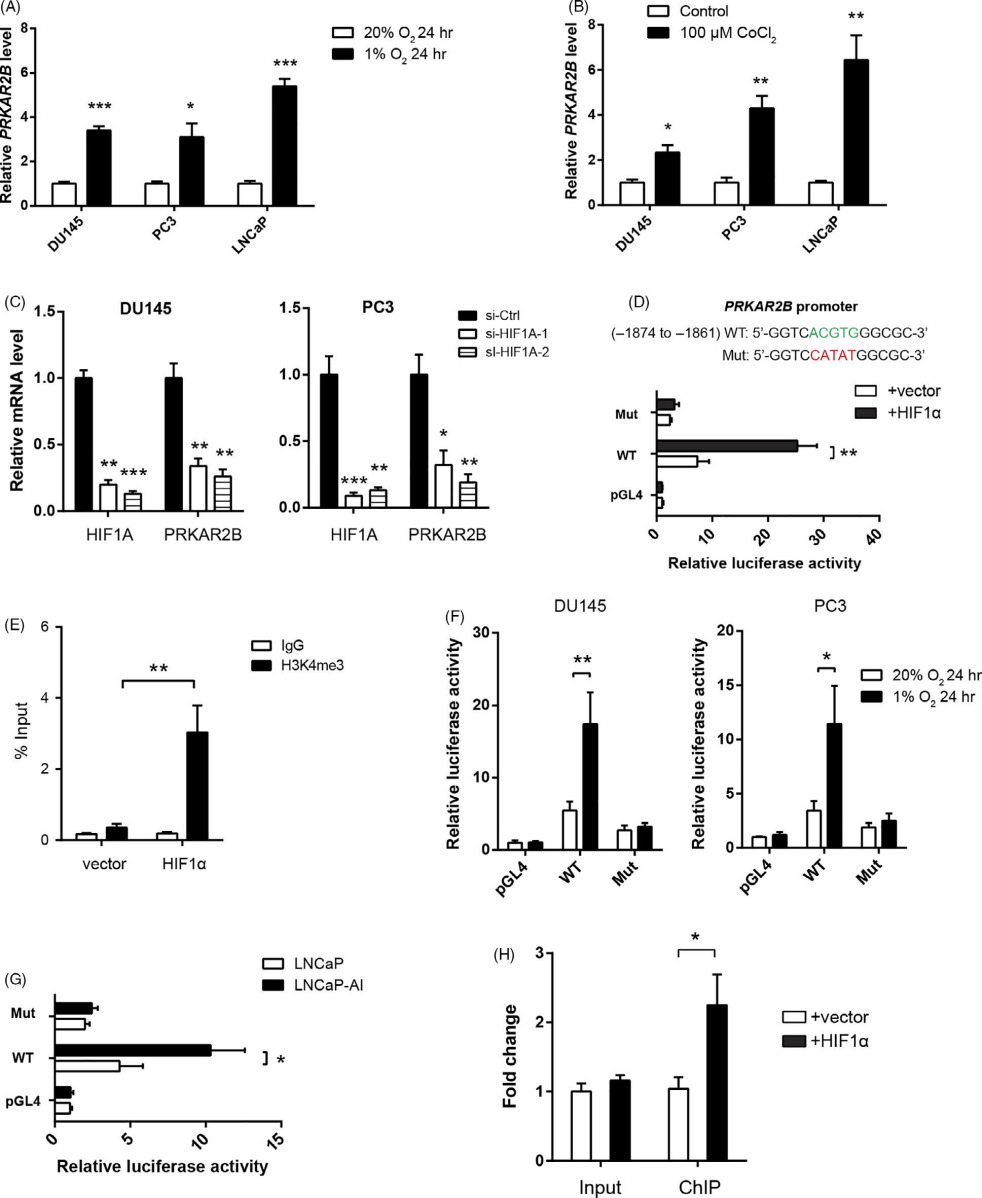
Transcriptional regulation of PRKAR2B by HIF‐1α in prostate cancer. A, Real‐time qPCR analysis of PRKAR2B expression in DU145, PC3, and LNCaP cells under hypoxia (1% O_2_) and normoxia (20% O_2_) condition. B, Real‐time qPCR analysis of PRKAR2B expression in DU145, PC3, and LNCaP cells after 100 μmol/L CoCl_2_ treatment for 24 h. C, Effect of HIF‐1α knockdown on the PRKAR2B expression in DU145 and PC3 cells. D, Luciferase activity of PRKAR2B gene promoter reporters in DU145 cells transfected with HIF‐1α or empty vector. Red sites represents the putative HIF‐1α‐binding sites; Mut, mutant; WT, wild‐type. E, PRKAR2B ChIP‐PCR for control input and H3K4me3 ChIP. F, The PRKAR2B gene promoter activity in DU145 and PC3 cells under hypoxia (1% O_2_) and normoxia (20% O_2_) condition. G, The PRKAR2B gene promoter activity in LNCaP and LNCaP‐AI cells. H, PRKAR2B ChIP‐PCR for control input and HIF‐1α ChIP. *P < .05; **P < .01; ***P < .001

Hypoxia is a constant characteristic of prostate tumour microenvironment, and HIF‐1α is the key regulator of the transcriptional response to hypoxic stress.^24^ Given the close correlation between PRKAR2B and HIF‐1α, we hypothesized that PRKAR2B might be induced by hypoxia in prostate cancer. To test this hypothesis, we cultured DU145, PC3 and LNCaP cells under both normoxic and hypoxic condition for 24 hours As a result, PRKAR2B expression was drastically induced by hypoxia in three tested cell lines (Figure 5A). Using a chemical inducer of HIF‐1α, CoCl_2_, we also obtained similar results (Figure 5B). Moreover, knockdown of HIF‐1α significantly reduced PRKAR2B expression in DU145 and PC‐3 cells (Figure 5C). To further confirm this observation, we first examined 2 kb of promoter regions of PRKAR2B gene for putative HIF‐1α response element (5′‐ACGTG‐3′). Secondly, we generated a promoter reporter containing the putative binding sites (ACGTG) (Figure 5D). As a result, HIF‐1α increased the reporter activity of PRKAR2B, while mutation of the binding site largely abolished HIF‐1α‐induced promoter reporter activity (Figure 5D). Trimethylation of histone H3 at lysine 4 (H3K4me3) is one of the universal markers of an active promoter. As shown in Figure 5E. A significant increase in H3K4me3 was observed in the promoter region of PRKAR2B. Moreover, the reporter activity was induced by hypoxia in DU145 and PC3 cells (Figure 5F) and LNCaP‐AI cells had higher reporter activity than LNCaP cells (Figure 5G). To further confirm this observation, we performed chromatin immunoprecipitation (CHIP) experiment. ChIP and input cycle threshold values were normalized separately to empty vector control as 1. Consistently, ChIP result revealed that HIF‐1α can interact directly with PRKAR2B gene promoters (Figure 5H). Taken together, these data above demonstrate that PRKAR2B expression can be induced by hypoxia and indicate a positive PRKAR2B‐HIF‐1α feedback loop in prostate cancer.

### 3.5 The growth‐promoting role of PRKAR2B in prostate cancer is glycolysis‐dependent

**FIGURE 6:**
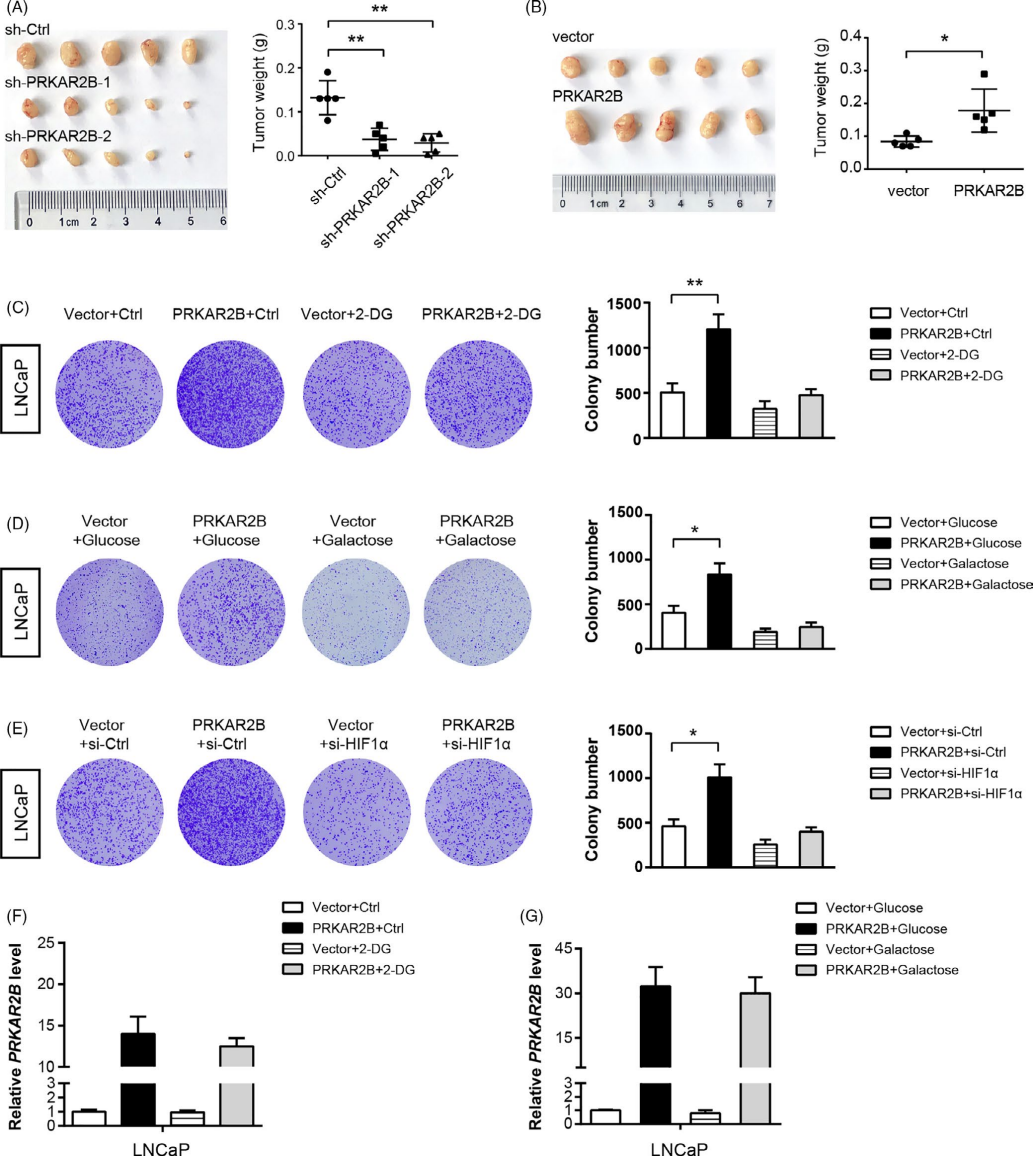
The growth‐promoting role of PRKAR2B in prostate cancer is glycolysis‐dependent. A, In vivo growth assay of that sh‐Ctrl and sh‐PRKAR2B DU145 cells. B, In vivo growth assay of that ov‐vector and PRKAR2B‐overexressing LNCaP cells. C, Plate clone formation assay showed the effect of PRKAR2B on LNCaP cell proliferation in the presence or absence of 5 mmol/L 2‐DG. D, Plate clone formation assay showed the effect of PRKAR2B on LNCaP cell proliferation in the culture medium containing 25 mmol/L glucose or 25 mmol/L galactose. E, Measurement of the effect of PRKAR2B overexpression on LNCaP cell proliferation in the presence or absence of HIF‐1α knockdown. F, Real‐time qPCR analysis of PRKAR2B expression in DU145 and PC3 cells after 2‐DG treatment. G, Real‐time qPCR analysis of PRKAR2B expression in DU145 and PC3 cells after galactose treatment. *P < .05; **P < .01

Previously, we have revealed that PRKAR2B contributes to tumour growth in prostate cancer.^11^ Using a subcutaneous xenograft model, we examined the in vivo effects of PRKAR2B‐overexpressing and PRKAR2B knockdown on prostate cancer cells. The result showed that PRKAR2B knockdown significantly retarded xenograft tumour growth of DU145 cells, while PRKAR2B overexpression promoted xenograft tumour growth of LNCaP cells (Figure 6A,B). Because glycolysis can enable tumour growth by providing intermediates for biosynthesis and NADPH production, we tested whether glycolysis is needed for PRKAR2B‐mediated growth advantage. Firstly, we treated LNCaP cells with 5 mmol/L 2‐Deoxy‐d‐glucose (2‐DG), a well‐known glycolysis inhibitor. As expected, 2‐DG largely compromised the growth advantage induced by PRKAR2B overexpression in LNCaP cells (Figure 6C). Secondly, we replaced glucose with galactose (which has a much lower rate than glucose entry into glycolysis) in the culture medium and performed plate clone formation experiments. The result showed that galactose also abrogated the tumour‐promoting role of PRKAR2B in cell proliferation (Figure 6D). Moreover, we also determined the oncogenic role of PRKAR2B in the presence or absence of HIF‐1α knockdown. As shown in Figure 6C, PRKAR2B significantly promoted cell proliferation in control but not HIF‐1α knockdown LNCaP cells (Figure 6E). By cell viability assay and cell apoptosis assay (Figure S5), we confirmed similar phenomenon. By real‐time qPCR, we examined the PRKAR2B expression upon treatment of 2‐DG or galactose. Notably, 2‐DG or galactose had no significant effect on PRKAR2B expression in LNCaP cells (Figure 6F,G). Given that PRKAR2B promotes aerobic glycolysis in PCa cells and blocking glycolysis largely abrogates the tumour‐promoting effect of PRKAR2B, we conclude that PRKAR2B‐induced Warburg effect may be essential for prostate tumorigenesis.

## 4 DISCUSSION

In the current study, we investigated the possible regulatory of PRKAR2B in the Warburg metabolism of human PCa. Our results showed that (a) PRKAR2B enhances PCa cell glycolysis, (b) PRKAR2B is able to upregulate HIF‐1α protein level, (c) HIF‐1α transcriptionally induces PRKAR2B expression in PCa; (d) PRKAR2B‐mediated tumour growth in PCa cells is glycolysis‐dependent. These findings decipher a novel role of PRKAR2B in PCa and provide a new opportunity to develop therapeutic strategy for PCa treatment.

Emerging evidences indicate that the Warburg effect is largely regulated by loss of tumour suppressors or activation of oncogenes, such as TP53, KRAS and Myc.^25^, ^26^ Blocking the Warburg effect in cancer cells leads to reduced tumourigenicity and increased chemosensitivity, suggesting that targeting the Warburg metabolism is a promising for developing cancer treatment.^27^ PRKAR2B is overexpressed in CRPC mouse models and patients, and knockdown of PRKAR2B significantly inhibits CRPC cell proliferation, invasion and survival by mainly modulates cell cycle gene expression.^11^ Notably, PRKAR2B can also activate Wnt/β‐catenin signalling pathway to promote the epithelial‐mesenchymal transition process and prostate cancer metastasis.^13^ In this study, we for the first time uncovered a new role of PRKAR2B in regulating cancer cell glycolysis. This finding further broadens our insight into the oncogenic activities of PRKAR2B in prostate cancer. Moreover, tumour growth induced by PRKAR2B is largely glycolysis‐dependent indicative of the critical contribution of PRKAR2B in PCa glycolysis. In mice, PRKAR2B deficiency increases energy expenditure by inducing the expression of uncoupling protein 1 and other thermogenic genes, limited weight gain, and improved glucose metabolism through a mechanism involving increased PKA activity.^28^ Previously, PKA was shown to modulate HIF1α by phosphorylation and stimulate its transcriptional activity.^29^ Consistently, we revealed that inhibition of PKA activity with H89 suppressed HIF‐1α protein expression and glycolytic metabolism in PCa cells. Therefore, our findings open the way of using specific PKA inhibitors to PCa treatment by targeting HIF‐1α and inhibiting the Warburg effect. However, whether altered PKA activity is involved in the underlying mechanism by which PRKAR2B promote aerobic glycolysis warrants further investigation.

Adaptation of cancer cells to a hypoxic tumour microenvironment is of great importance for their malignant growth and distant metastasis.^30^ One of the major mechanisms mediating the hypoxic response is induction of HIF‐1α expression, which controls reprogramming of energy metabolism by direct targeting metabolism‐related genes.^31^ Here we revealed that PRKAR2B can facilitate tumour glycolysis by increasing HIF‐1α, which is commonly overexpressed in prostate cancer. As a key transcription factor for aerobic glycolysis, HIF‐1α initiates the transcription of SLC2A1, PFKP, PKM and LDHA mRNA. As a result, increased transcripts lead to high protein expression of these glycolytic components, which ultimately promote aerobic glycolysis in PCa. Increased HIF‐1α expression in PCa is closely correlated with rapid cell proliferation and higher metastatic potential.^32^ Importantly, HIF‐1α expression has been demonstrated to increase as prostate cancer progressed from androgen‐dependent to androgen‐independent states.^33^ Consistent with this notion, we found that LNCaP‐AI cells have higher HIF‐1α level than its parental LNCaP cells. Interestingly, we further identified that HIF‐1α regulates PRKAR2B expression at transcriptional level. Importantly, tumour growth advantage induced by PRKAR2B could be largely abrogated by HIF‐1α knockdown, suggesting the importance of PRKAR2B‐HIF‐1α loop in PCa development.

In conclusion, we have shown that dysregulated PRKAR2B is involved in the altered metabolism in PCa. We further demonstrate a reciprocal regulation between PRKAR2B and HIF‐1α and this loop have a critical role in the Warburg effect and tumour growth. Interference of PRKAR2B‐HIF‐1α loop may hold promise for preventing PCa development.

## CONFLICT OF INTEREST

The authors declare that there is no conflict of interests.

## AUTHOR CONTRIBUTIONS

Lei Xia, Jian Sun, Shaowei Xie and Chenfei Chi performed the experiments. Baijun Dong, Yiran Huang, Weiliang Xia, Jianjun Sha and Wei Xue provided key intellectual input in the conception and design of these studies. Yinjie Zhu and Jiahua Pan analysed the data. All authors reviewed the manuscript.

## Supporting information

Appendix S1

## Data Availability

The data generated or analysed during this study are included in this article.

## References

[1] Siegel R , Ma J , Zou Z , Jemal A . Cancer statistics, 2014. Cancer J Clin. 2014;64(1):9‐29. 10.3322/caac.21208 24399786

[2] Wong YN , Ferraldeschi R , Attard G , de Bono J . Evolution of androgen receptor targeted therapy for advanced prostate cancer. Nat Rev Clin Oncol. 2014;11:365‐376. 24840076 10.1038/nrclinonc.2014.72

[3] Katzenwadel A , Wolf P . Androgen deprivation of prostate cancer: leading to a therapeutic dead end. Cancer Lett. 2015;367:12‐17. 26185001 10.1016/j.canlet.2015.06.021

[4] Halabi S , Dutta S , Tangen CM , et al. Overall survival of Black and White men with metastatic castration‐resistant prostate cancer treated with docetaxel. J Clin Oncol. 2019;37:403‐410. 30576268 10.1200/JCO.18.01279 PMC6804881

[5] Ingrosso G , Detti B , Scartoni D , et al. Current therapeutic options in metastatic castration‐resistant prostate cancer. Semin Oncol. 2018;45:303‐315. 30446166 10.1053/j.seminoncol.2018.10.001

[6] Froehner M , Wirth MP . Enzalutamide in metastatic prostate cancer before chemotherapy. N Engl J Med. 2014;371:424‐433. 25354112 10.1056/NEJMc1410239

[7] Pattabiraman DR , Bierie B , Kober KI , et al. Activation of PKA leads to mesenchymal‐to‐epithelial transition and loss of tumor‐initiating ability. Science. 2016;351(6277):aad3680. 26941323 10.1126/science.aad3680 PMC5131720

[8] Yoon H , Jang H , Kim EY , et al. Knockdown of PRKAR2B results in the failure of oocyte maturation. Cell Physiol Biochem. 2018;45:2009‐2020. 29518769 10.1159/000487978

[9] Peverelli E , Ermetici F , Corbetta S , et al. PKA regulatory subunit R2B is required for murine and human adipocyte differentiation. Endocr Connect. 2013;2:196‐207. 24145613 10.1530/EC-13-0049 PMC3847920

[10] Elliott MR , Tolnay M , Tsokos GC , Kammer GM . Protein kinase A regulatory subunit type II beta directly interacts with and suppresses CREB transcriptional activity in activated T cells. J Immunol. 2003;171:3636‐3644. 14500661 10.4049/jimmunol.171.7.3636

[11] Sha J , Xue W , Dong B , et al. PRKAR2B plays an oncogenic role in the castration‐resistant prostate cancer. Oncotarget. 2017;8:6114‐6129. 28008150 10.18632/oncotarget.14044 PMC5351617

[12] Xia L , Han Q , Chi C , et al. Transcriptional regulation of PRKAR2B by miR‐200b‐3p/200c‐3p and XBP1 in human prostate cancer. Biomed Pharmacother. 2020;124:109863. 31986411 10.1016/j.biopha.2020.109863

[13] Sha J , Han Q , Chi C , et al. PRKAR2B promotes prostate cancer metastasis by activating Wnt/beta‐catenin and inducing epithelial‐mesenchymal transition. J Cell Biochem. 2018;119:7319‐7327. 29761841 10.1002/jcb.27030

[14] Cantor JR , Sabatini DM . Cancer cell metabolism: one hallmark, many faces. Cancer Discov. 2012;2:881‐898. 23009760 10.1158/2159-8290.CD-12-0345 PMC3491070

[15] Jiang SH , Li J , Dong FY , et al. Increased serotonin signaling contributes to the warburg effect in pancreatic tumor cells under metabolic stress and promotes growth of pancreatic tumors in mice. Gastroenterology. 2017;277‐291.e19. 10.1053/j.gastro.2017.03.008 28315323

[16] Gonthier K , Poluri RTK , Audet‐Walsh E . Functional genomic studies reveal the androgen receptor as a master regulator of cellular energy metabolism in prostate cancer. J Steroid Biochem Mol Biol. 2019;191:105367. 31051242 10.1016/j.jsbmb.2019.04.016

[17] Geng H , Xue C , Mendonca J , et al. Interplay between hypoxia and androgen controls a metabolic switch conferring resistance to androgen/AR‐targeted therapy. Nat Commun. 2018;9:4972. 30478344 10.1038/s41467-018-07411-7 PMC6255907

[18] Audet‐Walsh E , Dufour CR , Yee T , et al. Nuclear mTOR acts as a transcriptional integrator of the androgen signaling pathway in prostate cancer. Genes Dev. 2017;31:1228‐1242. 28724614 10.1101/gad.299958.117 PMC5558925

[19] Wang J , Xu W , Wang B , et al. GLUT1 is an AR target contributing to tumor growth and glycolysis in castration‐resistant and enzalutamide‐resistant prostate cancers. Cancer Lett. 2020;485:45‐55. 32428663 10.1016/j.canlet.2020.05.007

[20] Yu T , Yang G , Hou Y , et al. Cytoplasmic GPER translocation in cancer‐associated fibroblasts mediates cAMP/PKA/CREB/glycolytic axis to confer tumor cells with multidrug resistance. Oncogene. 2017;36:2131‐2145. 27721408 10.1038/onc.2016.370

[21] Palorini R , De Rasmo D , Gaviraghi M , et al. Oncogenic K‐ras expression is associated with derangement of the cAMP/PKA pathway and forskolin‐reversible alterations of mitochondrial dynamics and respiration. Oncogene. 2013;32:352‐362. 22410778 10.1038/onc.2012.50

[22] Gagliano SA , Tiwari AK , Freeman N , et al. Protein kinase cAMP‐dependent regulatory type II beta (PRKAR2B) gene variants in antipsychotic‐induced weight gain. Hum Psychopharmacol. 2014;29:330‐335. 24737441 10.1002/hup.2407

[23] Tang Z , Kang B , Li C , Chen T , Zhang Z . GEPIA2: an enhanced web server for large‐scale expression profiling and interactive analysis. Nucleic Acids Res. 2019;47:W556‐W560. 31114875 10.1093/nar/gkz430 PMC6602440

[24] Semenza GL . HIF‐1 mediates metabolic responses to intratumoral hypoxia and oncogenic mutations. J Clin Investig. 2013;123:3664‐3671. 23999440 10.1172/JCI67230 PMC3754249

[25] Ying H , Kimmelman AC , Lyssiotis CA , et al. Oncogenic Kras maintains pancreatic tumors through regulation of anabolic glucose metabolism. Cell. 2012;149:656‐670. 22541435 10.1016/j.cell.2012.01.058 PMC3472002

[26] Hanahan D , Weinberg RA . Hallmarks of cancer: the next generation. Cell. 2011;144:646‐674. 21376230 10.1016/j.cell.2011.02.013

[27] Jones RG , Thompson CB . Tumor suppressors and cell metabolism: a recipe for cancer growth. Genes Dev. 2009;23:537‐548. 19270154 10.1101/gad.1756509 PMC2763495

[28] Su J , Wu W , Huang S , et al. PKA‐RIIB deficiency induces brown fatlike adipocytes in inguinal WAT and promotes energy expenditure in male FVB/NJ mice. Endocrinology. 2017;158:578‐591. 27967236 10.1210/en.2016-1581

[29] Bullen JW , Tchernyshyov I , Holewinski RJ , et al. Protein kinase A–dependent phosphorylation stimulates the transcriptional activity of hypoxia‐inducible factor 1. Science Signaling. 2016;9(430):ra56. 27245613 10.1126/scisignal.aaf0583 PMC5541497

[30] Zou C , Yu S , Xu Z , et al. ERRalpha augments HIF‐1 signalling by directly interacting with HIF‐1alpha in normoxic and hypoxic prostate cancer cells. J Pathol. 2014;233:61‐73. 24425001 10.1002/path.4329

[31] Semenza GL . Targeting HIF‐1 for cancer therapy. Nat Rev Cancer. 2003;3:721‐732. 13130303 10.1038/nrc1187

[32] Thomas R , Kim MH . HIF‐1 alpha: a key survival factor for serum‐deprived prostate cancer cells. Prostate. 2008;68:1405‐1415. 18563715 10.1002/pros.20808 PMC2593855

[33] Zhong H , Agani F , Baccala AA , et al. Increased expression of hypoxia inducible factor‐1alpha in rat and human prostate cancer. Can Res. 1998;58:5280‐5284. 9850048

