# Supplementary material for: PRKAR2B‐HIF‐1α loop promotes aerobic glycolysis and tumour growth in prostate cancer
Source: Cell Prolif. 2020 Oct 7;53(11):e12918. doi: 10.1111/cpr.12918 (PMC7653268; doi:10.1111/cpr.12918)
Supplement: Supplementary file 1 — Appendix S1 [file CPR-53-e12918-s001.docx]

**Supplementary File**

**Supplementary Methods**

Tissue sections were deparaffinized in xylene and rehydrated in graded alcohol. Antigen retrieval was done in tris-ethylenediaminetetraacetic acid (EDTA) pH 9.0 buffer at 95 °C for 20 min. Tissue sections were then incubated in Tris-buffered saline (TBS) for 5 min. Endogenous peroxidase blocking was performed in 3% H_2_O_2_ for 10 min. Subsequently, tissue sections were incubated in primary antibody against PRKAR2B (1:500, PA5-28266, Invitrogen) overnight at 4 °C. The slides were washed for 5 times with 0.01 M phosphate-buffered saline for 10 min. After that, tissue sections were incubated with horseradish peroxidase (HRP) tagged secondary antibody for 45 min at room temperature. Subsequently, the tissue sections were washed in PBS and stained with 3,3-diaminobenzidine (DAB). Finally, the slides were counterstained, dehydrated, and mounted.

**Supplementary Figures**

**
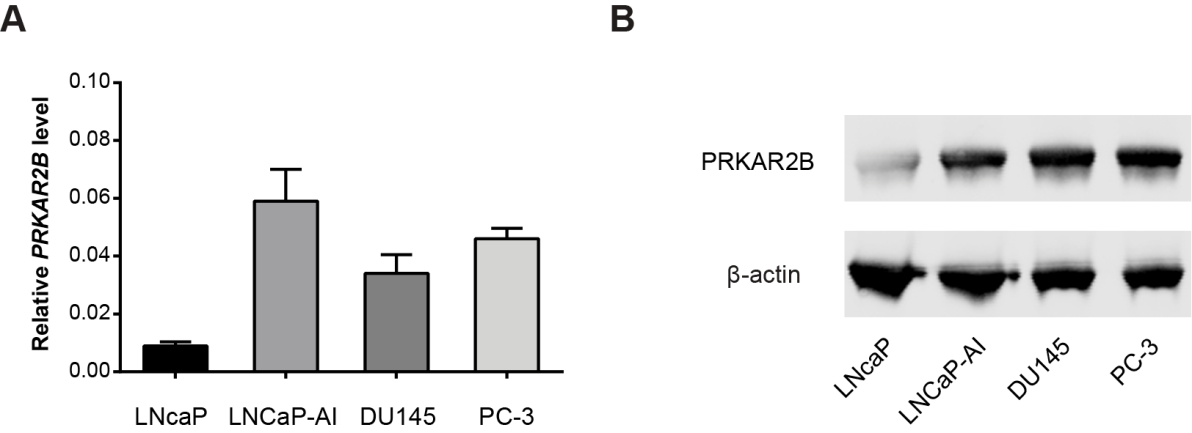
**

**Supplementary Fig. 1 PRKAR2B expression in prostate cancer. (A)** Real-time qPCR analysis of PRKAR2B mRNA expression in PCa cell lines. **(B)** Western blotting analysis of PRKAR2B protein expression in PCa cell lines.

**
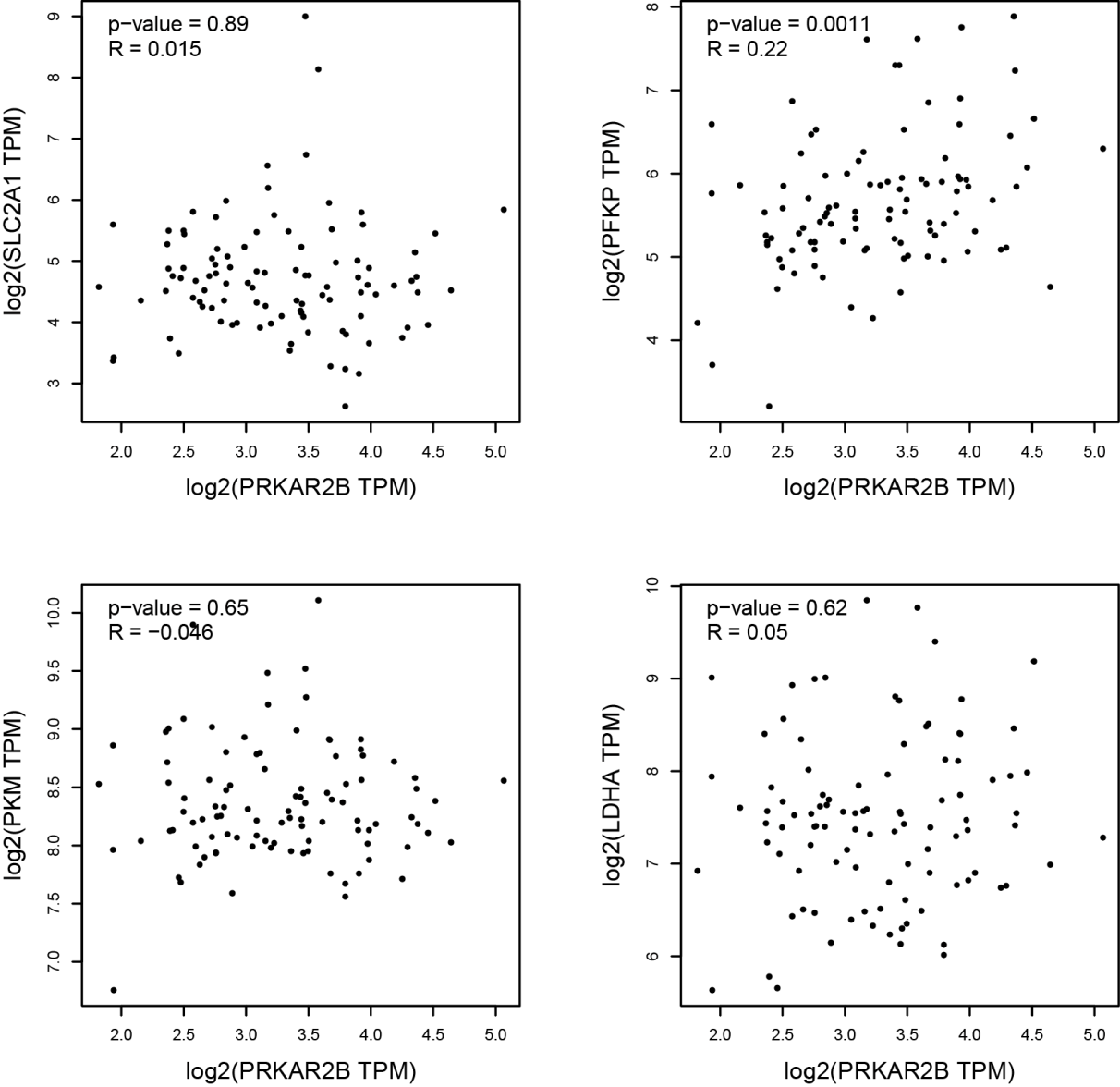
**

**Supplementary Fig. 2 Correlation analysis between PRKAR2B expression and glycolytic components in normal prostate tissues (n = 52).** Data were obtained from the GTEx cohort analyzed by the online GEPIA2 database (http://gepia2.cancer-pku.cn/#index).

**
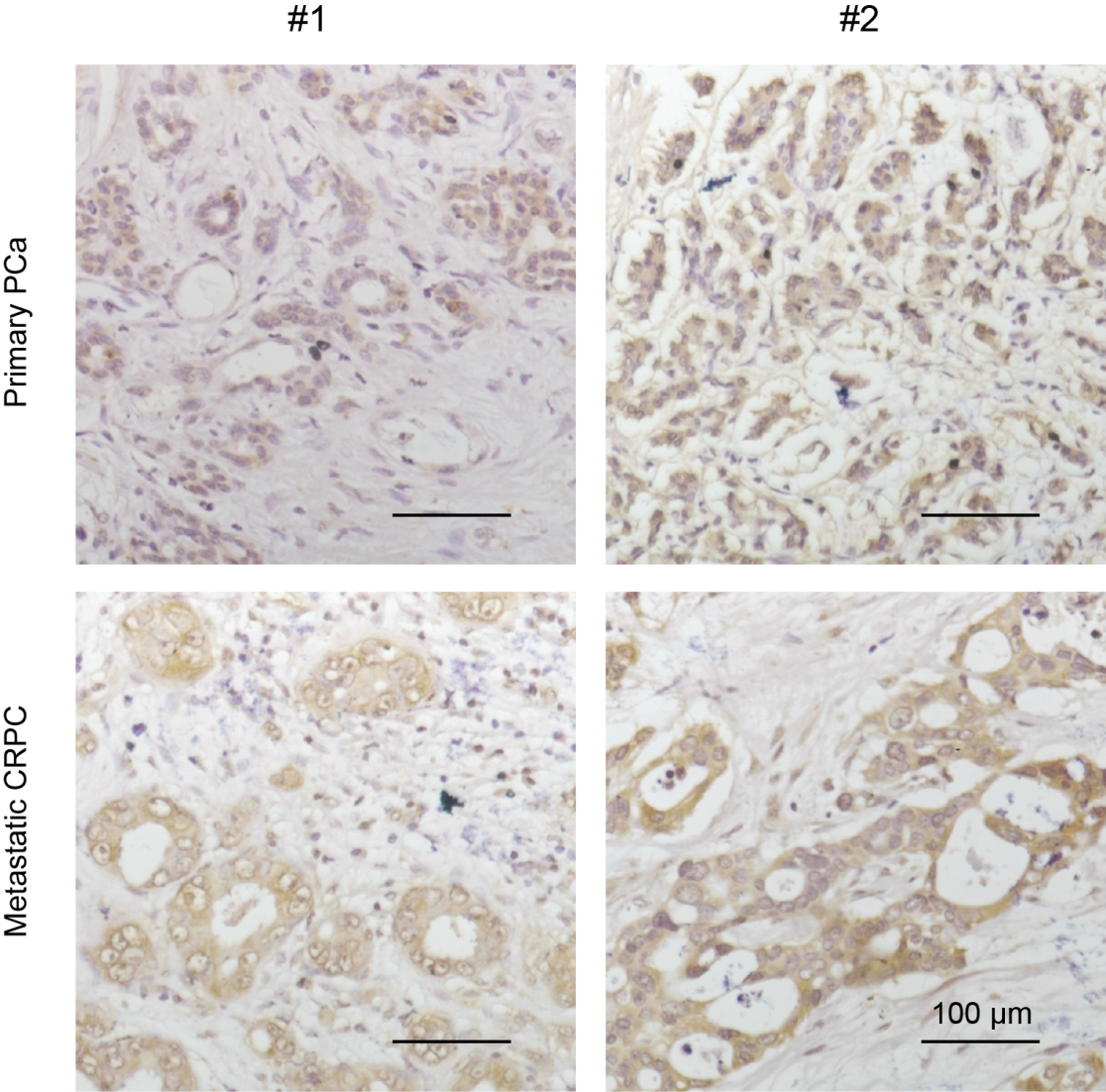
**

**Supplementary Fig. 3 IHC analysis of PRKAR2B expression in PCa tissues.** Representative immunohistochemistry images for PRKAR2B expression in primary PCa tissues and 17 metastatic CRPC tissues. Scale bar: 100 μm.

**
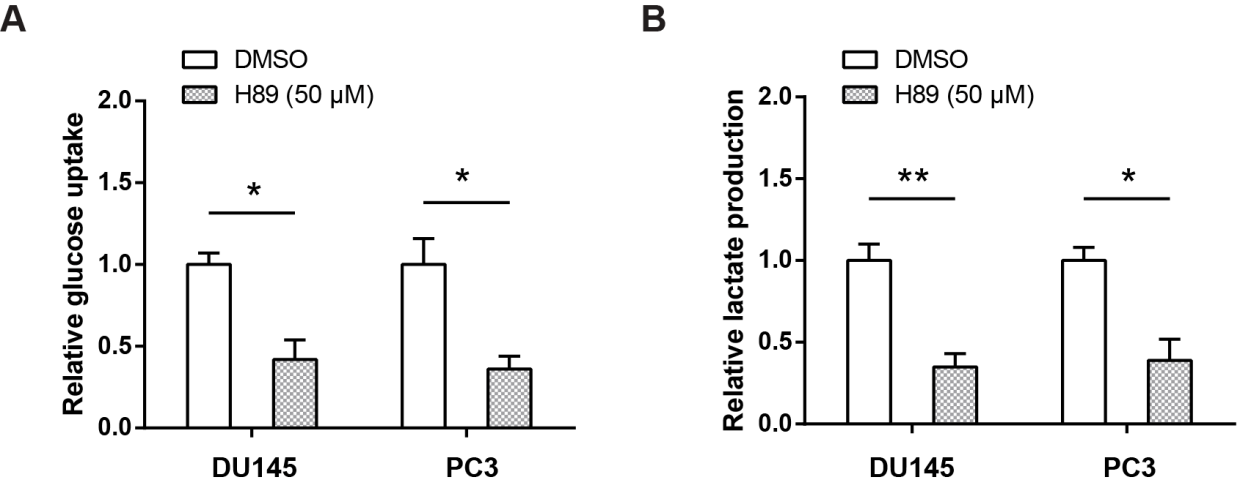
**

**Supplementary Fig. 4 PKA inhibition blocks PCa glycolysis. (A)** Measurement of glucose uptake in DU145 and PC3 cells upon H89 (50 μM) treatment for 24 h. **(B)** Measurement of lactate production in DU145 and PC3 cells upon H89 (50 μM) treatment for 24 h. *p < 0.05; **p < 0.01.

**
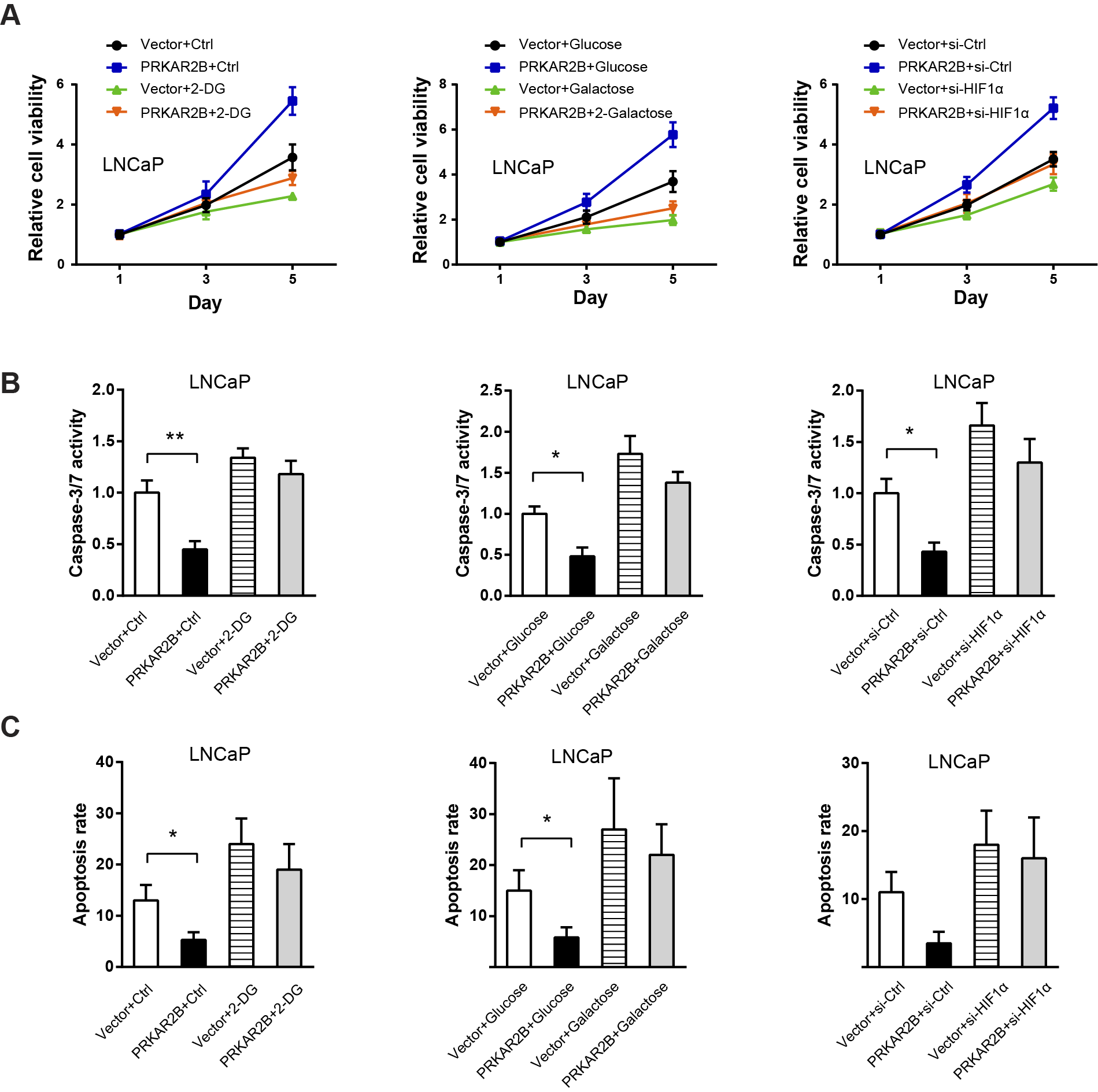
**

**Supplementary Fig. 5 PRKAR2B promotes cell proliferation and avoids cell apoptosis in a glycolysis-dependent manner. (A)** Measurement of the effect of PRKAR2B overexpression on LNCaP cell viability by CCK-8 assay in the presence or absence of 2-DG, galactose, or HIF-1α knockdown. **(B-C)** Measurement of the effect of PRKAR2B overexpression on starvation-induced LNCaP cell apoptosis by Caspase-3/7 assay or Annexin V/PI staining assay in the presence or absence of 2-DG, galactose, or HIF-1α knockdown. *p < 0.05; **p < 0.01.
